# Quantitative Kinetic Analyses of Shutting Off a Two-Component System

**DOI:** 10.1128/mBio.00412-17

**Published:** 2017-05-16

**Authors:** Rong Gao, Ann M. Stock

**Affiliations:** Department of Biochemistry and Molecular Biology, Center for Advanced Biotechnology and Medicine, Rutgers University—Robert Wood Johnson Medical School, Piscataway, New Jersey, USA; University of Washington

**Keywords:** PhoBR, autoregulation, computer modeling, histidine kinase, phosphatases, systems biology, transcriptional regulation, two-component regulatory systems

## Abstract

Cells rely on accurate control of signaling systems to adapt to environmental perturbations. System deactivation upon stimulus removal is as important as activation of signaling pathways. The two-component system (TCS) is one of the major bacterial signaling schemes. In many TCSs, phosphatase activity of the histidine kinase (HK) is believed to play an essential role in shutting off the pathway and resetting the system to the prestimulus state. Two basic challenges are to understand the dynamic behavior of system deactivation and to quantitatively evaluate the role of phosphatase activity under natural cellular conditions. Here we report a kinetic analysis of the response to shutting off the archetype *Escherichia coli* PhoR-PhoB TCS pathway using both transcription reporter assays and *in vivo* phosphorylation analyses. Upon removal of the stimulus, the pathway is shut off by rapid dephosphorylation of the PhoB response regulator (RR) while PhoB-regulated gene products gradually reset to prestimulus levels through growth dilution. We developed an approach combining experimentation and modeling to assess *in vivo* kinetic parameters of the phosphatase activity with kinetic data from multiple phosphatase-diminished mutants. This enabled an estimation of the PhoR phosphatase activity *in vivo*, which is much stronger than the phosphatase activity of PhoR cytoplasmic domains analyzed *in vitro*. We quantitatively modeled how strong the phosphatase activity needs to be to suppress nonspecific phosphorylation in TCSs and discovered that strong phosphatase activity of PhoR is required for cross-phosphorylation suppression.

## IMPORTANCE

Activation of TCSs has been extensively studied; however, the kinetics of shutting off TCS pathways is not well characterized. We present comprehensive analyses of the shutoff response for the PhoR-PhoB system that reveal the impact of phosphatase activity on shutoff kinetics. This allows development of a quantitative framework not only to characterize the phosphatase activity in the natural cellular environment but also to understand the requirement for specific strengths of phosphatase activity to suppress nonspecific phosphorylation. Our model suggests that the ratio of the phosphatase rate to the nonspecific phosphorylation rate correlates with TCS expression levels and the ratio of the RR to HK, which may contribute to the great diversity of enzyme levels and activities observed in different TCSs.

## INTRODUCTION

Bacteria often respond to diverse environmental perturbations through two-component systems (TCSs) that involve a conserved phosphotransfer between a histidine kinase (HK) sensor and a cognate response regulator (RR) (1, 2). The HK possesses autokinase, phosphotransferase, and, often, phosphatase activities that regulate the phosphorylation level of its cognate RR. Activation of the pathway occurs through stimulus-mediated control of the kinase activity and/or the phosphatase activity, leading to a change of the RR phosphorylation level and modulation of the output response, most commonly, transcription regulation. There is great diversity in the response dynamics, regulatory mechanisms, and design architectures among different TCSs. Understanding the general principles of regulation, such as why some HKs possess a strong phosphatase activity and why expression levels of TCS proteins are different in different systems, requires specifying the kinetic parameters of TCS activities. Measurement of these parameters has traditionally been performed using reconstituted systems with purified proteins outside the cellular context. Here we designed an approach to evaluate these parameters using *in vivo* measurements.

Despite extensive research on activation dynamics of various TCSs, much less is known about the cellular events of system deactivation upon removal of the stimulus. How quickly is an RR dephosphorylated *in vivo*? What is the time scale for resetting an RR-regulated response, e.g., gene expression, to prestimulus levels? The flux of phosphate (Pi) and the expression of response genes are both under dynamic control. For simplicity, we use “shutoff” to describe all the cellular events that take place upon stimulus removal.

The first step in shutting off the system is RR dephosphorylation via alteration of the kinase activity and/or the phosphatase activity. In some systems, such as the noncanonical chemotaxis system, the RR is dephosphorylated by an auxiliary phosphatase (3, 4). However, many TCSs contain bifunctional HKs with opposing autokinase and RR phosphatase activities. It is believed that the intrinsic phosphatase activity in these TCSs provides a rapid dephosphorylation mechanism to shut off the system and to restore it to the original state once the stimulus is removed (1, 5). It has also been shown that the phosphatase activity can suppress cross-phosphorylation of RRs from noncognate HKs and maintain the system in an OFF state (6–9). Most studies have been focused on the static ON (high level of RR phosphorylation) and OFF (low level of RR phosphorylation) states of the pathway, and TCSs are often portrayed as simple switches that can toggle between these static states. Several studies have begun to explore the sophisticated control that TCS pathways can exert on signaling dynamics, with many focusing on system activation (10–15). However, it is also of great interest to understand the temporal dynamics of shutting off the pathway. Specifically, how quickly is a system turned off once the stimulus is removed, and how does phosphatase activity impact the kinetics of the shutoff response?

One determining factor for the shutoff dynamics is the kinetics of RR dephosphorylation. RR dephosphorylation by its cognate HK has been characterized *in vitro* for many TCSs (16–21). Except for a few reports on a cytoplasmic HK (20, 21), most studies have used either truncated or reconstituted membrane HK proteins to dephosphorylate phosphorylated RRs (RR~Ps) (16–19), raising questions about the integrity of these proteins and the accuracy of *in vitro* kinetics. Moreover, many HKs displayed relatively weak phosphatase activity, with half-lives of dephosphorylation ranging from several minutes to as long as half an hour (16–19). High concentrations of HK domains have been routinely used to observe significant RR dephosphorylation, while the ratio of HK to RR is usually much lower *in vivo* than those used *in vitro* (12, 22–24). This even led to the questioning of the physiological importance of such weak phosphatase activity for the HK EnvZ (25), although EnvZ has been shown to be capable of suppressing cross-phosphorylation *in vivo* (7). How faithfully *in vitro* conditions recapitulate the *in vivo* environment of TCS proteins is always a central issue. Therefore, it is important to evaluate phosphatase activity in its natural environment and to examine the kinetics of the shutoff response.

**FIG 1:**
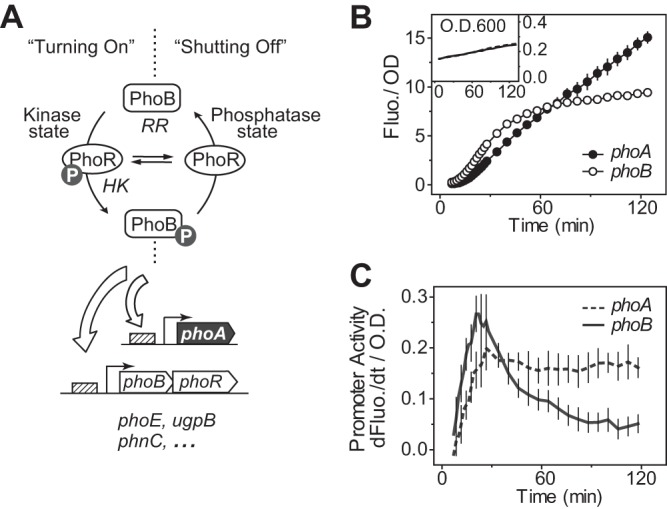
Different activation dynamics of PhoB-regulated promoters. (A) Schematic diagram of the PhoR-PhoB two-component pathway. (B and C) Temporal dynamics of YFP reporter activation illustrated by OD-normalized fluorescence (Fluo.) (B) and the first derivative of fluorescence (dFluo./dt) (C). WT strain BW25113 carrying reporter plasmid pRG161 (*phoA*) or pJZG202 (*phoB*) was assayed for Pi starvation responses. The first derivative of fluorescence measures the rate of YFP synthesis, reflecting the promoter activities. Error bars represent standard deviations (SDs) of data from 11 individual wells from a microplate assay. Data from different microplate assays show similar patterns of reporter activation dynamics.

Here we report an *in vivo* study of the shutoff kinetics of the *Escherichia coli* PhoR-PhoB TCS using both reporter assays and phosphorylation analyses. The PhoR-PhoB system regulates the assimilation of phosphorus in response to limitation of environmental Pi concentrations (Fig. 1A) (26, 27). Under Pi-depleted conditions, the HK PhoR catalyzes increased phosphorylation of PhoB, promoting regulation of a number of genes responsible for Pi uptake and utilization of different phosphorus sources. Under Pi-replete conditions, cells prefer Pi as the phosphorus source and the PhoBR activity is low. Deletion of the regulator gene *phoU* leads to hyperactivation of the PhoBR pathway in *E. coli* and is deleterious, possibly due to excessive accumulation of intracellular Pi or phosphate metabolites (27–29). Activation of the pathway has been well characterized *in vivo* (10, 23). Steady-state levels of phosphorylated PhoB (PhoB~P) and reporter activities of several PhoB-regulated genes have been cross-examined at different *phoBR* expression levels, allowing evaluation of the faithfulness of different reporters as measures of PhoB phosphorylation (10). Phosphatase activity has been demonstrated for a cytoplasmic fragment of PhoR *in vitro*; however, the activity is low, with a dephosphorylation half-life longer than 10 min (23, 30). We discovered that PhoB~P dephosphorylation occurred *in vivo* much more quickly than had been observed *in vitro*. Analyses of the shutoff response allow a more accurate estimation of the intrinsic phosphatase activity of PhoR, and the approach developed here can be extended to other TCSs.

## RESULTS

### Different reporters have distinct temporal profiles of promoter activation.

RR phosphorylation levels or TCS activities *in vivo* are usually inferred from transcription of downstream reporter genes. However, gene transcription often takes place under complicated control tailored for specific functions, sometimes involving multiple transcription regulators or feedback loops, resulting in different timings of activation or complex temporal patterns (13, 31). Thus, careful examination of whether a particular reporter can reflect the phosphorylation activities of TCSs is required. Because TCS activation is easier to observe than deactivation, activation kinetics of yellow fluorescent protein (YFP) reporters were examined initially for five different PhoB-regulated genes to identify a suitable reporter for PhoB phosphorylation (Fig. 1; see also Fig. S1 in the supplemental material). When cells were resuspended in Pi-depleted media, growth rates slowed and both *phoA* and *phoB* promoters were activated. The temporal profiles of these promoters were distinct thereafter. The rate of fluorescence increase was reduced for the *phoB-yfp* reporter at later time points, leading to a gradual plateauing of fluorescence. In contrast, the YFP level for the *phoA-yfp* reporter continued to show an almost linear increase throughout the assay time (Fig. 1B).

10.1128/mBio.00412-17.2 FIG S1  Temporal dynamics of YFP reporter activation illustrated by OD-normalized cellular fluorescence (A) and the first derivative of fluorescence (B). WT strains carrying plasmid pJZG202 (*phoB*), pRG347 (*phoE*), pRG346 (*ugpB*), pRG161 (*phoA*), or pRG162 (*phnC*) were assayed for Pi starvation response. Data represent averages of 11 individual wells for each reporter strain. Solid lines represent smoothed data calculated from adjacent averages. Download FIG S1, PDF file, 0.1 MB. Copyright © 2017 Gao and Stock. 2017 Gao and Stock This content is distributed under the terms of the Creative Commons Attribution 4.0 International license.

The fluorescence value at one particular time point reports all of the accumulated output of promoter activity prior to that time point. The time derivative of YFP fluorescence that indicated the rate of YFP synthesis and decay, with the latter presumed to be minor, was used to assess the real-time promoter activities and to represent the temporal profile. Most promoters tested displayed clear differences in their temporal responses (Fig. 1C; Fig. S1). Promoter activity of *phoB* peaked at ~20 min and slowly decreased afterward, consistent with a previously observed repression of the *phoB* promoter at higher PhoB~P concentrations (10). The *phoA* reporter reached a steady level 30 min after induction, agreeing with a steady PhoB phosphorylation level observed before (10). Thus, the *phoA* reporter was chosen for further analyses of its correlation with phosphorylation kinetics.

### Temporal profiles of *phoA* promoter activity correlate with phosphorylation kinetics.

**FIG 2:**
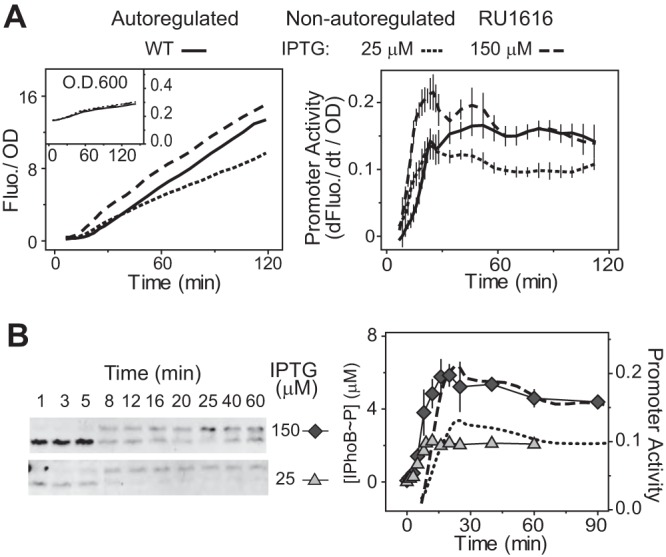
Correlation between promoter activities and phosphorylation kinetics. (A) Temporal dynamics of *phoA-yfp* activation in the autoregulated WT strain and the nonautoregulated RU1616 strain. IPTG concentrations of 25 μM and 150 μM were used to produce steady PhoB levels at ~2.4 μM and ~8.1 μM (23), respectively. Error bars represent SDs of data from at least 11 individual wells. (B) Time course of *in vivo* PhoB phosphorylation. Phosphorylation percentages quantified from Phos-tag gels (left) were used to calculate the concentrations of phosphorylated PhoB (right) by multiplying by the total PhoB expression levels. Error bars represent SDs of data from at least three independent experiments, and unseen error bars are smaller than symbol sizes. Dashed and dotted lines represent smoothed promoter activity data.

Because expression of PhoB is autoregulated in the wild-type (WT) strain, PhoB phosphorylation levels are difficult to quantify at early stages of activation due to low levels of PhoB. To better investigate the relation between promoter profiles and phosphorylation kinetics, the *phoA-yfp* reporter was placed in RU1616, a strain engineered with *phoBR* under control of a *lac* promoter to express PhoB/PhoR proteins at constant levels independently of the stimulus (23). An IPTG (isopropyl-β-d-thiogalactopyranoside) concentration of 150 μM has been shown to produce a PhoB level in RU1616 comparable to the steady-state level of autoregulated PhoB in the WT strain under Pi-depleted conditions, leading to similar steady levels of promoter activities for RU1616 and the WT strain ~60 min after induction (Fig. 2A). A small extent of overshoot kinetics, or impulse response (32, 33), was observed for RU1616 but not for the WT strain, with promoter activities reaching a high level before relaxing to the eventual steady state. Impulse responses have been suggested to result from negative feedback in TCS pathways (12, 15, 34), but the reason for the absence of the overshoot kinetics in the WT strain is not clear. Nevertheless, the temporal profile nicely recapitulates the PhoB phosphorylation kinetics *in vivo* (Fig. 2B). There is an interval of 6 to 8 min between the phosphorylation and promoter responses, possibly due to the time required for transcription, translation, and YFP maturation. RU1616 grown with a lower IPTG concentration of 25 μM displayed lower promoter activities, consistent with its low PhoB and PhoB~P levels (Fig. 2B). The closely matched phosphorylation and promoter profiles suggest that *phoA-yfp* is a reliable reporter for *in vivo* phosphorylation kinetics.

### The PhoR-PhoB system is shut off rapidly upon stimulus removal.

**FIG 3:**
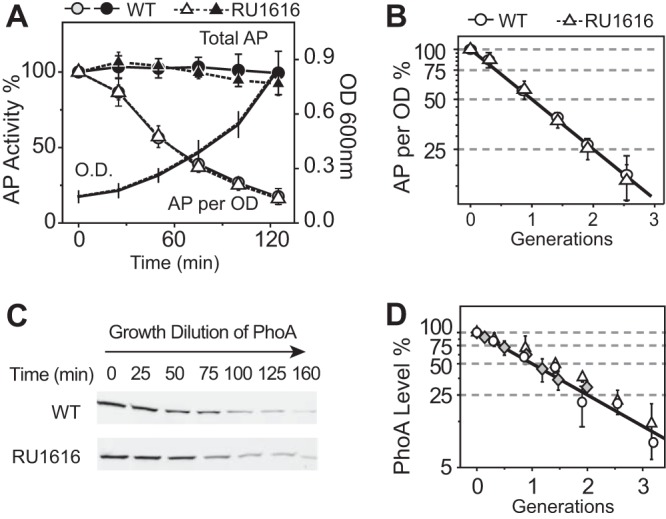
Growth dilution of PhoA upon stimulus removal. Alkaline phosphatase activities (A and B) and immunoblots of PhoA protein (C and D) were monitored after Pi-starved cells were resuspended in Pi-replete media. Initial AP and PhoA protein levels at time zero were set as 100%. Open symbols represent AP activities or PhoA levels from identical amounts of cells (0.3 OD ⋅ ml) harvested at the indicated times. Multiplying these OD-normalized data by bacterial OD values yields the total AP activities (solid symbols). An IPTG concentration of 150 μM was always present in the media for RU1616 to maintain a steady PhoB level close to the WT PhoB concentration under Pi-depleted conditions. Growth ODs were further converted to bacterial doubling generations by exponential fitting of the growth curve to replot the dependence of PhoA on bacterial growth (B and D). RU1616 and the WT strain have similar doubling times of around 45 min. Solid lines in panels B and D represent the theoretical growth dilution curves. Gray diamonds represent the data from RU1616 with a different doubling time (55 min) due to the change of nutrients in MOPS media (D). Data are shown as means ± SDs of results from at least three independent experiments.

The endogenous *phoA* gene encodes an alkaline phosphatase (AP) whose activity and protein concentration have been routinely used for reporter assays. Cells resumed growth when bacteria were moved from Pi-depleted media to Pi-replete media, while optical density (OD)-normalized AP activities and PhoA expression levels decreased (Fig. 3A and C). The WT strain and the nonautoregulated RU1616 strain displayed identical temporal profiles of responses, suggesting that positive autoregulation has little impact on the shutoff kinetics. Interestingly, the total amount of AP activity from the whole culture remained constant. The decrease in the AP level per cell is simply due to the OD increase, i.e., growth dilution. OD-normalized AP activities, or protein levels, are halved for every doubling of bacterial growth (Fig. 3B and D). When the doubling time was changed from 45 min to 55 min, the AP concentrations followed the same growth dilution curve (Fig. 3D). Because alkaline phosphatase is relatively stable (27), the constant level of total AP indicates that there was no new synthesis of AP protein once the stimulus was removed. The data imply that the signaling system had been shut off rapidly, preventing extraneous production of AP protein.

**FIG 4:**
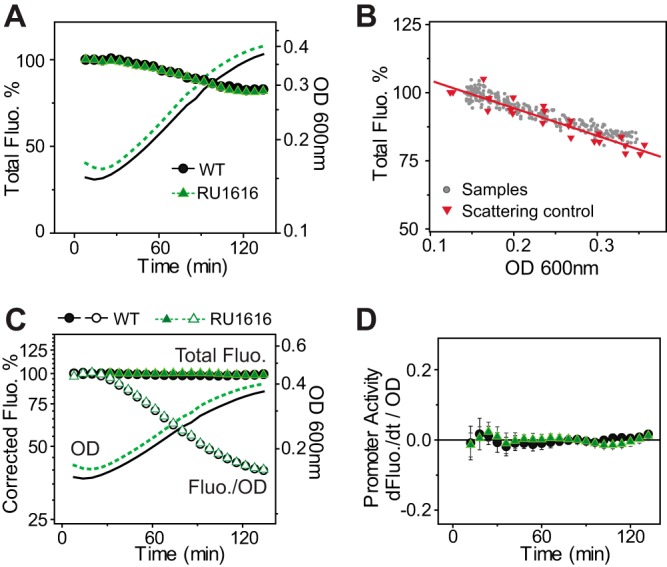
Temporal dynamics of *phoA*-*yfp* reporter fluorescence upon stimulus removal. (A) Raw data of total fluorescence after resuspension of Pi-starved cells in Pi-replete media (1 mM Pi). An IPTG concentration of 150 μM was used to maintain a steady PhoB level for RU1616. Initial fluorescence was arbitrarily set as 100%. (B) Reduction of fluorescence with increasing OD of bacteria. Gray circles represent data from individual wells examined in the experiment described in the panel A legend, and red triangles represent fluorescence of identical amounts of pure YFPs mixed with different ODs of bacteria. The solid line indicates an empirical linear correlation for fluorescence correction based on data from pure YFP samples. (C) Time course of corrected fluorescence. Scattering or inner-filter effects due to bacterial OD were corrected for total fluorescence (solid symbols), and corrected fluorescence values were divided by OD values to give OD-normalized data (open symbols). (D) Shutoff of promoter activities. Data are shown as means ± SD of results from 22 individual wells.

Fluorescence of the *phoA-yfp* reporter was measured with high temporal resolution to examine the fast shutoff response when bacteria were resuspended in Pi-replete media. Surprisingly, unlike the constant level of total PhoA activity, total fluorescence gradually decreased along with the increase of bacterial OD (Fig. 4A). The decrease was not due to repeated exposures to excitation light, which can potentially photobleach the fluorophore (Fig. S2). It was reasoned that the relatively high turbidity of bacterial cultures may reduce the intensity of excitation light and/or emission light by light scattering because the size of bacteria (0.5 to 1 μm) is on the same scale as the wavelengths of excitation and emission light (488 nm and 530 nm). To correct for the scattering effect or other potential absorbance-related inner-filter effects caused by increased density of bacteria, identical amounts of purified YFPs were mixed with increasing ODs of nonfluorescent bacteria for fluorescence calibration. High ODs of bacteria indeed lowered the fluorescence reading, and an empirical correlation between fluorescence and OD was used for fluorescence correction (Fig. 4B). Corrected total fluorescence appeared to be constant throughout the assay (Fig. 4C), and the promoter activities were shut off almost completely within the first 15 min (Fig. 4D), consistent with the responses determined by analysis of the AP activities.

10.1128/mBio.00412-17.3 FIG S2  Constant YFP fluorescence after continuous reading. Bacteria with constitutive expression of YFP (BW25141/pRG278) were resuspended in chloramphenicol-containing MOPS media (100 μg/ml) with ODs of 0.03 and 0.08 followed by repeated readings performed once per minute (black circles). Control wells contained identical amounts of bacteria but were read only three times at 0, 16, and 32 min (red circles). Frequent reading, or frequent exposures to excitation light, did not significantly alter the total fluorescence of cells, suggesting that there was no significant photobleaching of YFP under experimental conditions. Download FIG S2, PDF file, 0.1 MB. Copyright © 2017 Gao and Stock. 2017 Gao and Stock This content is distributed under the terms of the Creative Commons Attribution 4.0 International license.

**FIG 5:**
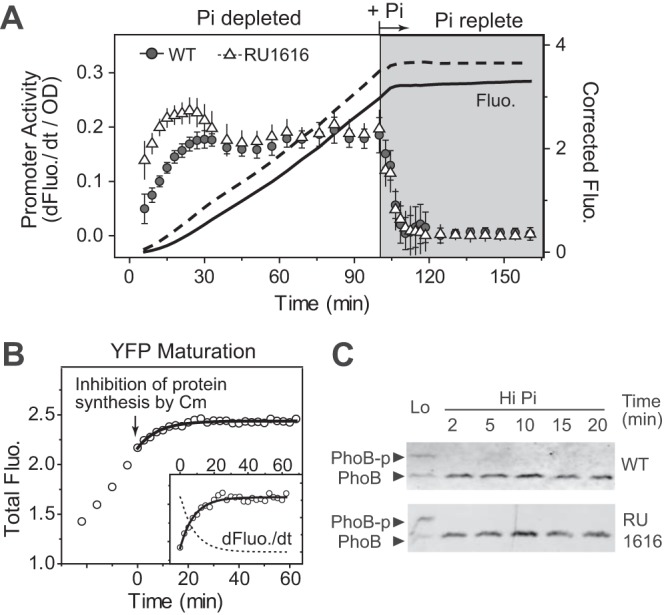
Dependence of reporter shutoff dynamics on YFP maturation and dephosphorylation kinetics. (A) Activation and shutoff of promoter activities. Pi was added directly to Pi-starved cultures to reach 1 mM to turn off the pathway. Data are shown as means ± SD of results from 22 individual wells. (B) YFP maturation kinetics. *phoA-yfp* was activated by Pi starvation, and protein synthesis was inhibited by 320 μg/ml of chloramphenicol, as indicated by the arrow. Data shown represent averages of results from 10 wells. The solid line shows the exponential fit with a half-time of 5.5 min. The first derivative illustrated in the inset was calculated from the fitted curve. (C) Phos-tag analyses of PhoB dephosphorylation kinetics. Results from one experiment representative of three independent experiments are shown.

Extra time required for bacteria resuspension, plate loading, and preparation precluded data readings for the first several minutes of the shutoff response. To monitor the initial period of reporter responses, Pi was directly added to Pi-starved cultures during a kinetic assay and the temporal dynamics of shutoff were measured after the steady state of activation had been reached (Fig. 5A). Upon Pi addition, fluorescence of both WT and RU1616 continued to increase for ~10 min and became stable afterward. Accordingly, the first derivative of fluorescence (dFluo./dt) decreased to zero in ~10 min and the half-time, the time required to reach half of the maximal level, is 4 min.

The increase of fluorescence might have been due to the presence of newly expressed YFPs and/or the maturation of YFPs produced before the addition of Pi. The maturation time of YFP was thus determined by measuring the fluorescence kinetics after protein synthesis was inhibited by addition of chloramphenicol (Cm) (Fig. 5B). The half-time of YFP maturation is 5.5 min, very close to the 4-min half-time for the reporter shutoff dynamics. Changing the YFP reporter to a cyan fluorescent protein (CFP) reporter caused the half-time to become ~9 min, matching the maturation half-time of CFP (Fig. S3). Thus, the increase of fluorescence is attributed to fluorescent protein maturation rather than production of new fluorescent proteins, suggesting that the pathway is turned off almost instantaneously upon Pi addition. This suggestion is supported by *in vivo* analyses of PhoB phosphorylation (Fig. 5C). Phosphorylated PhoB was no longer present 2 min after Pi addition.

10.1128/mBio.00412-17.4 FIG S3  Comparison of shutoff dynamics with CFP maturation kinetics. (A to C) Decreases in promoter activities due to Pi addition (open symbols) or inhibition of protein synthesis by erythromycin (solid symbols) were compared for RU1823 (A), RU1825 (B), and RU1826 (C). An IPTG concentration of 5 μM was used for steady expression of PhoB. (D and E) CFP maturation kinetics appeared to be almost identical for three strains. Exponential fit of the promoter activities analyzed as described for panel D (Ery-treated samples) gives maturation half-times similar to those determined for the following strains: RU1823 (WT), 9.2 min; RU1825 (PhoR^T217M^), 8.4 min; RU1826 (PhoB^F20D^), 8.8 min. Averaging the three sets of data gives the maturation half-time for CFP: 8.8 min. Data are shown as means ± SD of results from 11 individual wells. Download FIG S3, PDF file, 0.1 MB. Copyright © 2017 Gao and Stock. 2017 Gao and Stock This content is distributed under the terms of the Creative Commons Attribution 4.0 International license.

### Phosphatase activity affects the dynamics of shutoff responses.

To examine how the phosphatase activity impacts the shutoff kinetics, we constructed two mutants in the PhoR-PhoB TCS with potentially distinct mechanisms for diminished phosphatase activities. One carries an F20D mutation in PhoB, and the other has a T217M mutation in PhoR (23, 27, 35). The F20D mutation is distant from the phosphorylation site and has been suggested to weaken the binding affinity between PhoR and PhoB by ~8-fold (23). The T217 residue, conserved in the HisKA family of HKs, is close to the autophosphorylated histidine residue and has been suggested to position a nucleophilic water molecule for phosphatase activity (5). Phosphorylation profiling for the activation response revealed that PhoR^T217M^ reached higher steady-state PhoB~P levels than the WT at high expression levels of PhoBR (Fig. S4A). Fitting performed with a phosphorylation cycle model (23, 36) suggested that the T217M mutation most likely caused an ~4.5-fold decrease of *k*_*p*_, the phosphatase catalytic rate constant (Fig. S4; see also Text S1 in the supplemental material).

10.1128/mBio.00412-17.5 FIG S4  Phosphorylation profiling of RU1825 (PhoR^T217M^). (A) Phosphorylation levels (upper) and total expression levels (lower) of PhoB were analyzed at different IPTG concentrations. Samples were harvested ~90 min after Pi depletion and prepared as described before (23). (B) Dependence of PhoB~P levels on PhoB concentrations. Total PhoB concentrations were determined as described previously (10). Phosphorylation levels were calculated as the product of total PhoB concentration and phosphorylation percentages derived from Phos-tag gels. Circles and triangles represent data from two independent experiments. The solid line indicates the best fit with the model shown in panel C, while the dashed line represents the simulated WT phosphorylation determined using crossing point (*Cp*) and threshold cycle (*Ct*) values determined previously (23). Michaelis-Menten kinetic parameters were used to describe the phosphotransfer and dephosphorylation reactions, while the steady-state levels of PhoB~P were determined using the composite parameters *Cp* and *Ct* (see Text S1). It appears that PhoR^T217M^ has a *Ct* value similar to that of the WT but a larger *Cp* value, resulting in a higher steady-state phosphorylation level than that seen with the WT. The high value of *Cp* is unlikely to be due to an increased *K*_*m*_ because the change of affinity between PhoB and PhoR would simultaneously alter both *Cp* and *Ct*. Given the position of the T217 residue and its conserved role in phosphatase activity in the HisKA family of proteins (5), an ~4.5-fold-higher *Cp* for PhoR^T217M^ is most consistent with an ~4.5-fold-lower *k*_*p*_. Download FIG S4, PDF file, 0.6 MB. Copyright © 2017 Gao and Stock. 2017 Gao and Stock This content is distributed under the terms of the Creative Commons Attribution 4.0 International license.

10.1128/mBio.00412-17.1 TEXT S1  Model of PhoB phosphorylation and dephosphorylation. Download TEXT S1, PDF file, 0.2 MB. Copyright © 2017 Gao and Stock. 2017 Gao and Stock This content is distributed under the terms of the Creative Commons Attribution 4.0 International license.

**FIG 6:**
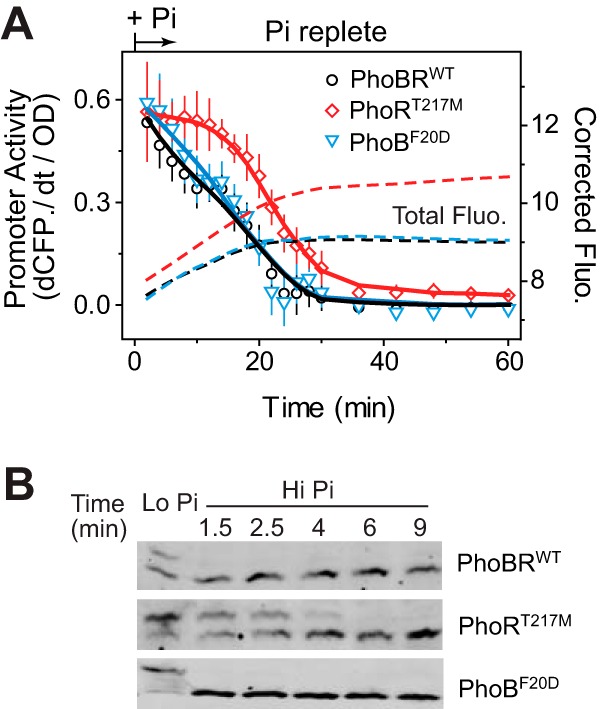
Shutoff dynamics of phosphatase mutants. (A) Promoter activities of the *phoA-cfp* reporter in strains RU1823 (WT), RU1825 (PhoR^T217M^), and RU1826 (PhoB^F20D^). A Cm-resistant CFP reporter plasmid (pRG381) was used instead of pRG161 to avoid conflicts with antibiotics in the strains described above. An IPTG concentration of 5 μM was used to maintain a steady PhoB level at ~11 μM (10). Bacteria had been Pi starved for 70 min before direct addition of Pi to the cultures, and the time of Pi addition was set as time zero. Dashed lines represent the corrected total fluorescence, and solid lines represent smoothed promoter activity data. Data are shown as means ± SD of results from 11 individual wells. (B) Time course of PhoB dephosphorylation.

To measure the response dynamics of the mutants upon Pi addition, a CFP reporter plasmid was used to avoid conflicts between YFP reporter plasmid pRG161 and the mutant strains with respect to resistance to antibiotics. The first derivatives all decreased upon Pi addition (Fig. 6A) with kinetics determined by effects of both PhoB dephosphorylation and CFP maturation. When PhoB dephosphorylation is completed very shortly after Pi addition, the kinetics of fluorescence derivatives are dominated by the slower CFP maturation kinetics. The first derivatives for the PhoB^F20D^ strain and the WT decreased at a rate similar to the CFP maturation rate, indicating an immediate shutoff upon stimulus removal (Fig. S3). In contrast, the PhoR^T217M^ strain responded more slowly than the WT, displaying a biphasic decrease of fluorescence derivatives affected by both dephosphorylation and CFP maturation kinetics. The first derivatives remained relatively high for several minutes initially, indicating significant promoter activity that results from residual PhoB~P caused by slow PhoB dephosphorylation. Afterward, it followed a gradual decreasing trend similar to that of CFP maturation kinetics, suggesting that PhoR^T217M^ is capable of shutting off the majority of promoter activity. Dephosphorylation kinetics *in vivo* were consistent with the dynamics of promoter activities (Fig. 6B). Phosphorylated PhoB was completely gone within 1.5 min for the WT and PhoB^F20D^ strains, while it took 6 min for PhoR^T217M^ to complete dephosphorylation. Clearly, diminished phosphatase activity in PhoR^T217M^ caused a delay in dephosphorylation and the promoter activity remained active during the initial several minutes when a significant amount of PhoB~P was still present.

### Kinetic model of RR dephosphorylation.

**FIG 7:**
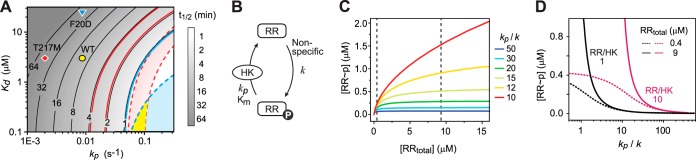
Model and simulation of PhoB dephosphorylation. (A) Contour plot of the dephosphorylation half-time derived from a Michaelis-Menten model with parameters *K*_*d*_ and *k*_*p*_. Parameters for the WT (circle) (*K*_*d*_ = 3 μM and *k*_*p*_ = 0.0087 s^−1^) were derived from previous *in vitro* analyses, and PhoB^F20D^ (triangle) has been suggested to have an 8-fold-higher *K*_*d*_ (23). The phosphorylation profiling shown in Fig. S4 suggested an ~4.5-fold-lower *k*_*p*_ for PhoR^T217M^ (diamond). Parameters derived *in vitro* all resulted in half-times much longer than those observed *in vivo*. The half-time for PhoB^F20D^ is less than 1 min *in vivo* (solid blue line), highlighting the parameter space for the WT (shaded in blue) on the basis of an 8-fold difference in *K*_*d*_. The half-time for PhoR^T217M^ is between 2 and 4 min (solid red lines), and an ~4.5-fold-lower *k*_*p*_ results in the potential WT parameters (shaded in red). The cross-section (shaded in yellow) represents the WT parameter space that can give PhoB^F20D^ a half-time of less than 1 min and PhoR^T217M^ a half-time between 2 and 4 min, assuming that the relative change of *K*_*d*_ and *k*_*p*_ remains constant for the mutants. (B) Model schematics of the TCS OFF state. Nonspecific RR phosphorylation is described by a first-order reaction with the rate constant *k*. The phosphatase activity of the cognate HK ensures that no significant amount of RR~P can accumulate in the absence of stimulus. (C and D) In the OFF state, steady-state levels of RR~P are dependent on the ratio of the phosphatase rate to the nonspecific phosphorylation rate constant (*k*_*p*_/*k*), the total concentration of RR (C), and the ratio of RR to HK (D). Vertical dashed lines in panel C represent the PhoB concentrations in the absence (left) or presence (right) of stimuli. A *K*_*m*_ of 0.3 μM was used for modeling based on *in vivo* estimations.

It is surprising that PhoB^F20D^ did not show any significant delay of *in vivo* response even though its weak affinity did result in slow dephosphorylation kinetics *in vitro* (23). To understand how the difference between phosphatase mutants impacts the shutoff dynamics, different effects on half-times of PhoB dephosphorylation have been modeled for different parameter values of the HK-RR dissociation constant (*K*_*d*_) and the phosphatase catalytic rate constant (*k*_*p*_) (Fig. 7A). Parameter values for the WT have been determined *in vitro* using a truncated cytoplasmic fragment of PhoR (23), giving a half-time longer than 10 min at the cellular concentrations of PhoB and PhoR. Diminishing the phosphatase activity through increasing the *K*_*d*_ by 8-fold for PhoB^F20D^ or decreasing the *k*_*p*_ by 4.5-fold for PhoR^T217M^ resulted in half-times longer than 30 min, which is not consistent with the observed *in vivo* dephosphorylation kinetics.

Apparently, the parameters determined *in vitro* did not truly reflect the enzyme activities *in vivo*. Our analyses of the shutoff response make the estimation of *in vivo* parameters possible. Strain PhoR^T217M^ displayed a dephosphorylation half-time between 2 and 4 min, which limits the parameter space for the WT, given a 4.5-fold difference in *k*_*p*_ (Fig. 7A, area shaded in red). On the other hand, dephosphorylation of PhoB^F20D^ suggested 1 min as an upper limit of the half-time. Lowering the *K*_*d*_ by 8-fold establishes an upper boundary for the WT parameter space (Fig. 7A, area shaded in blue). The cross-section of the two areas (Fig. 7A, area shaded in yellow) represents the possible parameter values for the WT with much larger *k*_*p*_ and smaller *K*_*d*_ values than those determined *in vitro*. These parameters indicate very strong phosphatase activity for the WT and a dephosphorylation half-time in the range of seconds. Such fast kinetics are beyond the temporal resolution of our *in vivo* analyses; thus, the WT appeared to have kinetics indistinguishable from those of PhoB^F20D^.

As shown in Fig. 6A, the promoter activity for PhoR^T217M^ did not decrease to zero. The residual activity led to basal expression higher than that of the WT, suggesting that strong phosphatase activity is required for maintaining the system in a completely OFF state. Phosphatase activity is known to suppress nonspecific phosphorylation from noncognate HKs or small-molecule phosphate donors (7–9, 37–39). Using the *in vivo* parameters estimated above, we modeled how the phosphatase activity impacts the steady-state level of RR~P in the presence of such a nonspecific phosphorylation influx (Fig. 7B to D). The RR~P level depends on the total concentration of RR as well as on the ratio of the phosphatase rate constant *k*_*p*_ to the nonspecific phosphorylation constant *k* (see details in Text S1). A high *k*_*p*_/*k* ratio indicates the presence of relatively strong phosphatase activity to ensure minimal RR phosphorylation even at high expression levels of RR. With a low *k*_*p*_/*k* ratio, a high concentration of RR results in significantly increased levels of RR~P.

For the autoregulated PhoR-PhoB system, dephosphorylation occurs rapidly once the stimulus is removed, but the RR concentration remains high (~9 μM) due to slow growth dilution. Thus, a low ratio of *k*_*p*_/*k* may not be sufficient to maintain a low PhoB~P level in the OFF state. It has been shown that low (3% to 5%) phosphorylation of PhoB in a *phoR* deletion strain still activates the downstream gene (23). Therefore, to maintain a PhoB~P level of less than 3% (approximately 0.27 μM), a ratio greater than 20 is required (Fig. 7C and D). The value of *k* is not known for PhoB but has been shown to be ~0.003 s^−1^ for cross talk between EnvZ and CpxR or between CpxA and OmpR (37). PhoB can be phosphorylated by CreC with comparable speeds (40). If a similar value of *k* is applicable to PhoB phosphorylation *in vivo*, a ratio of 20 would require a *k*_*p*_ of ~0.06 s^−1^, much larger than the value determined *in vitro* but right within the range estimated from our *in vivo* analyses. Moreover, the ratio of RR to HK is also important for the steady-state level of RR~P. A higher ratio of RR to HK indicates a relatively lower concentration of HK as the functional phosphatase; thus, stronger phosphatase activity, i.e., a higher ratio of *k*_*p*_/*k*, is required to maintain a RR~P level similar to that which exists in a system with a lower RR-to-HK ratio (Fig. 7D).

## DISCUSSION

Far from being simple ON and OFF switches, TCSs exert elaborate control on their activities for diverse regulatory tasks at different time scales. Control of when and how quickly a pathway is activated is often essential for the temporal order of gene expression and the success of environmental adaptation (10, 41, 42). The timely shutoff of a signaling pathway may be equally important because gratuitous gene expression in the absence of stimuli often confers fitness costs (43, 44). Understanding the behaviors of TCSs requires characterization of not only the wiring of the pathway but also the kinetic parameters of individual enzyme activities in their natural intracellular environments. We have previously reported a quantitative description of the kinase/phosphatase cycle during the activation of a TCS pathway (23). Here we have developed an approach for understanding the role of the phosphatase activity by examination of the shutoff kinetics.

Numerous studies have demonstrated that RRs can be phosphorylated by noncognate HKs and/or small molecules. While some TCSs are suggested to utilize cross-phosphorylation to integrate multiple signals (45), for many TCSs, signaling fidelity depends on the kinetic preference of phosphotransfer activity between cognate pairs and suppression of cross talk by the phosphatase activity of cognate HKs (6, 7, 46–49). Our model reveals how strong the phosphatase activity needs to be to suppress nonspecific phosphorylation. The steady-state RR~P level is a function of the total RR concentration, the ratio of RR to HK, and the ratio of the phosphatase rate to the nonspecific phosphorylation rate. A high expression level of RR, a high rate of nonspecific phosphorylation, and a high ratio of RR to HK all require strong phosphatase activity to maintain a low RR~P level in the OFF state. Our model establishes the quantitative correlation between architectural features of a TCS and phosphatase activity that would provide whatever basal RR~P level may be optimal for function in a particular system. It has been shown that the expression levels of TCSs or the ratios of RR to HK can differ greatly from one system to another (22–24, 50, 51). The great diversity of these properties among different TCSs may well correlate with different strengths of the intrinsic phosphatase activities of HKs.

Our model is a rather simple depiction of the system with an assumption that all the HK molecules are in a phosphatase state once a stimulus is removed. However, it is more likely that the stimulus changes the proportions of HK molecules in the phosphatase state or the kinase state. A fraction of the HK molecules may still possess kinase and phosphotransferase activities to phosphorylate the RR after the stimulus is removed. In this case, a phosphatase activity stronger than that predicted by our model is required to limit RR~P to a low level. A more complicated model with additional knowledge of the population of HK molecules in individual states is required for a thorough description of the system. Nevertheless, our simple model provides an estimation of the minimal phosphatase activity required for regulating cross-phosphorylation.

It is the ratio of the phosphatase rate to the nonspecific phosphorylation rate, rather than the phosphatase rate alone, that determines RR~P levels in the OFF state. Thus, it is necessary to assess the nonspecific phosphorylation rate to evaluate the strength of phosphatase activity required for cross talk suppression. A first-order reaction was used to approximate the rate of nonspecific phosphorylation from HK cross talk and/or small-molecule phosphate donors, such as acetyl phosphate. PhoB phosphorylation by acetyl phosphate has been shown to have a *K*_*m*_ of ~7 mM and a *k*_cat_ of ~0.003 s^−1^ (52). Given a cellular concentration of acetyl phosphate of 3 mM under some culture conditions (38), the rate constant *k* contributed by acetyl phosphate is ~0.001 s^−1^. As for HK cross talk, the affinity between noncognate TCS pairs is usually low, with the *K*_*d*_ of many pairs being greater than 55 μM (47), suggesting a *K*_*m*_ value much greater than the typical concentration of RRs. Thus, the Michaelis-Menten kinetics of cross-phosphorylation can be approximated with a first-order reaction. Cross talk between noncognate pairs, such as noncognate pair CreC and PhoB and noncognate pair PmrB and QseB in *E. coli*, HK08 and WalR in *Streptococcus pneumoniae*, or HrrS and ChrA in *Corynebacterium glutamicum*, has been reported to occur at comparable speeds, with half-times ranging from 3 to 10 min (8, 9, 39, 40), corresponding to values of *k* between 0.001 and 0.004 s^−1^. Combining the contributions from acetyl phosphate and HK cross talk, nonspecific phosphorylation appears to occur at a nontrivial rate with a value of *k* that can reach 0.005 s^−1^.

The phosphatase rate constant *k*_*p*_ determined *in vitro* for PhoR-PhoB is 0.0087 s^−1^, giving a ratio of *k*_*p*_/*k* much smaller than that required to keep PhoB~P at a low level. *In vivo* analyses of the shutoff response provided the boundary of kinetic parameter values, which is consistent with a much stronger phosphatase activity to maintain the pathway in the OFF state. The weak phosphatase activity observed *in vitro* may be attributed to a lack of regulation from the sensory domain that has been truncated or to a lack of repression from the Pi transporter proteins or the regulator protein PhoU. Because truncated HKs are widely used for *in vitro* characterization and because many display phosphatase activities similar to those shown by the cytoplasmic fragment of PhoR (16–18), underestimation of phosphatase activities may be a common issue in TCS studies.

The strong *in vivo* phosphatase activity of PhoR results in a remarkably fast shutoff response, with PhoB dephosphorylation completed in seconds. In contrast, the decrease in the level of PhoB-regulated PhoA is a consequence of growth dilution and occurs on a much longer time scale of hours. Interestingly, previous studies have shown that dephosphorylation of BvgA is completed within 15 min, which is also much less than the time (2 days) required for returning the induced BvgA protein level to the prestimulus level (11). Diminishing the phosphatase activity in PhoR^T217M^ delayed the response for only several minutes, a very small fraction of the time required for resetting the PhoA concentration. Thus, the exceptionally fast PhoB dephosphorylation kinetics may not provide significant advantages for resetting the baseline for PhoA levels but may rather be a product of the strong phosphatase activity selected by the need to suppress nonspecific phosphorylation. In a broader context, this suggests that for TCSs in which cross phosphorylation does not need to be minimized and thus might be expected to lack strong phosphatase activity, this lack would not greatly compromise shutoff of the system.

Although the possibility that fast dephosphorylation may be beneficial for the temporal dynamics of PhoB-regulated genes other than *phoA* cannot be excluded, there is clear physiological evidence that strong phosphatase activity of PhoR is essential for suppressing nonspecific phosphorylation to maintain the pathway in an OFF state under Pi-replete conditions. It has long been known that *phoR* mutants constitutively express PhoB-regulated genes at levels 20% to 25% of those observed in WT strains under conditions of phosphate deprivation (27, 53), and more recent studies have shown that *phoR* strains have low but significant levels of PhoB~P (23). Moreover, PhoR^T217M^, with only a few minutes’ delay in shutoff kinetics, was originally isolated for an elevated activity under Pi-replete conditions (35) and the reduced phosphatase activity appears to be no longer capable of fully suppressing cross phosphorylation. Modeling performed in this study has enabled estimation of the relatively high magnitude of phosphatase activity required to counterbalance nonspecific phosphorylation in the OFF state.

In addition to specific insights into the phosphatase activity of PhoR, the results from our studies illustrate approaches that may be broadly applicable to characterization of different TCSs. It is clear that fluorescence reporter assays can recapitulate RR phosphorylation or dephosphorylation kinetics *in vivo*. However, they can also be substantially influenced by other biophysical factors affecting fluorescence, such as scattering or maturation, or by additional regulation of specific promoters, such as repression of the *phoB* promoter. Great caution should be exercised in interpreting data from transcription reporter assays. Nevertheless, the temporal profiles of promoter activities can be used to examine different mutants and investigate the mechanisms of phosphatase activities. In this study, it was observed that mutation of the T217 residue in PhoR slows the shutoff response and reduces the catalytic rate constant of phosphatase; however, PhoR^T217M^ was still capable of dephosphorylating PhoB~P. These results suggest that either the conserved T217 residue is not indispensable for phosphatase activity or PhoB dephosphorylation can occur through alternative mechanisms *in vivo*, such as the reverse phosphotransfer from PhoB~P to PhoR and then to ADP. Furthermore, we have demonstrated that examination of different mutants may be used to provide estimation of *in vivo* kinetic parameters. In many cases, the reporter profile or *in vivo* phosphorylation dynamics for the WT system may not be sufficient to parameterize different activities of TCS proteins due to the complexity and limitations of transcription reporter and phosphorylation assays. Cross-examination of multiple mutants allows different parameter boundaries to be set for the WT system, leading to a more accurate estimation of *in vivo* kinetic parameters for better understanding the general principles of TCS regulation.

## MATERIALS AND METHODS

### Strains, plasmids, and growth conditions.

Strains and plasmids used in this study are listed in Table S1 in the supplemental material. DH5α was used for general cloning of plasmids, while all the strains used for *in vivo* assays were derived from BW25113. To examine the role of phosphatase in kinetic responses, a T217M mutation was introduced into *phoBR* by site-directed mutagenesis PCR using primers RG257 (5′-GCCATGAGTTACGTATGCCATTGACCGTG-3′) and RG258 (5′-CACGGTCAATGGCATACGTAACTCATGGC-3′). The fragment was cloned between the XbaI and SphI sites of pRG330, which contains a *trc* promoter, *lacI*^q^, and *phoB*^*F20D*^-*phoR*. The resulting pRG352 plasmid (as well as pRG330) was integrated into the chromosome of *phoBR*-deleted strain RU1621 using the conditional-replication, integration, and modular (CRIM) recombination strategies (54) to create RU1825 and RU1826, which allow IPTG-regulated expression of PhoB^F20D^ and PhoR^T217M^. For comparison, the pRG351 plasmid containing a nonmutated P_trc_-*phoBR* was created by cloning the XbaI and BsrGI fragment from pRG226 into pRG330 and was further integrated into RU1621 to create RU1823. Under conditions of 5 μM IPTG induction, both strains express PhoB at a level comparable to the stimulated WT level. Bacteria were grown at 37°C in LB broth or in morpholinepropanesulfonic acid (MOPS) minimal media with 0.4% glucose and amino acid mix (40 μg/ml) as described previously (10).

10.1128/mBio.00412-17.6 TABLE S1  Strains and plasmids used in this study. Download TABLE S1, PDF file, 0.1 MB. Copyright © 2017 Gao and Stock. 2017 Gao and Stock This content is distributed under the terms of the Creative Commons Attribution 4.0 International license.

### *In vivo* assays for determination of alkaline phosphatase, protein expression, and phosphorylation levels.

Cells from overnight MOPS cultures were used to inoculate fresh Pi-replete (1 mM KH_2_PO_4_) MOPS media. Once the OD reached 0.3 to 0.5, bacteria were harvested and resuspended in MOPS media again for phosphate starvation. In previous assays (10), an initial Pi concentration of 50 μM was used to allow sufficient bacterial growth from a starting OD of ~0.05 before Pi became depleted to the activation threshold. The exact timing of phosphate starvation is dependent on the Pi consumption rate and is difficult to determine. To overcome this kinetic uncertainty in this study, cell pellets from different strains were washed with MOPS medium (30 to 50 μM Pi, nonactivating) twice and directly resuspended in MOPS medium (2 μM Pi, activating) with a starting OD of ~0.15. The time of resuspension was designated the starting time of phosphate starvation. Bacteria were subsequently sampled at different time points for assaying activation response kinetics.

To examine the response kinetics of shutting off the system, bacterial cultures that had been phosphate starved for 1.5 to 2 h were washed with MOPS medium (0 μM Pi) once and resuspended in Pi-replete MOPS medium (1 mM Pi) with a starting OD of ~0.15 to ~0.2. Aliquots were removed at indicated time points for alkaline phosphatase assays and immunoblot analyses. Samples for PhoB phosphorylation analyses were prepared slightly differently. After the cultures had been phosphate starved for only 1.5 h, Pi was directly added to reach a final concentration of 1 mM followed by consecutive sampling up to 30 min after Pi addition. Bacteria pelleted before the Pi addition were used as samples representing 0 min.

For protein expression, *in vivo* phosphorylation, and AP activity analyses, aliquots from bulk cultures were removed at indicated time points and pelleted. AP activities were measured as described before (55) in the presence of ~7 mM substrate (*p*-nitrophenylphosphate), and the relative rates of absorbance change at 420 nm were used to represent the AP activities. Protein expression levels were measured using quantitative Western blotting and anti-AP (Sigma-Aldrich) or anti-PhoB. PhoB phosphorylation levels were measured with Phos-tag gel analyses as previously described (23). Cell pellets equal to approximately 0.3 OD ⋅ ml cells were lysed in 55 μl 1× BugBuster reagent (Novagen) followed by denaturation with 18 μl 4× SDS loading buffer. All samples were immediately flash-frozen in dry ice/ethanol and later analyzed using Phos-tag gels and quantitative Western blotting.

### Fluorescence reporter assays.

Bacterial cultures were grown using a method similar to that described above. Harvested bacteria pellets were resuspended in prewarmed MOPs media with a starting OD of ~0.15. The inoculated cultures were immediately transferred to black 96-well plates with a clear bottom (Nunc; Thermo Scientific) and grown in a Varioskan plate reader (Thermo Scientific) at 37°C with constant shaking (3 mm orbital, 240 rpm). More than 10 replicates were grown simultaneously for each individual strain or condition. The time of bacterial resuspension was recorded as time zero. Plate reading did not start immediately after resuspension because of the time required for plate loading, and an additional incubation of 3 to 5 min allowed for warming the plate before the first reading. Fluorescence for YFP (excitation, 488 nm; emission, 530 nm) or CFP (excitation, 430 nm; emission, 475 nm) and absorbance (OD at 600 nm) were repeatedly measured at various time intervals. Responses were usually measured at a short time interval of 1.5 to 3 min for the first 20 to 30 min, while longer intervals, e.g., 6 min, were used for the later stage of response kinetics. It was found that the use of different time intervals did not significantly change the trend of the response. To investigate the immediate response to shutting off the system, bacterial cultures were assayed for phosphate starvation responses in 96-well plates for 70 or 90 min before the addition of 15 μl of 20 mM KH_2_PO_4_ (final concentration, ~1 mM) to inactivate the signaling pathway. After a brief shaking period of 10 s, the fluorescence and OD were continuously monitored at indicated time intervals.

### Scattering and inner-filter correction of fluorescence.

When starved cells resume fast growth in Pi-replete media, the fluorescence responses are accompanied by a large increase in bacterial OD that can alter the fluorescence reading through scattering and/or absorbance-related inner-filter effects. To correct for these effects, purified YFP fusion proteins were diluted in MOPS medium to obtain fluorescence readings similar to those obtained for the reporter profiles. Different amounts of nonfluorescent bacteria from fresh MOPS cultures were mixed with identical YFP samples, with the final OD ranging between 0.12 and 0.35. Fluorescence and OD readings were discovered to follow an empirical linear correlation within the tested OD range. The average slope of the percentage of decrease in fluorescence was calculated from three independent experiments. Absolute values of fluorescence changes that occurred upon OD increase were added back to the fluorescence measurements for correction of reporter profiles. Similar correction schemes were applied to the CFP reporter profiles as well.

### Data processing of reporter profiles.

Fluorescence and OD readings were used to calculate the promoter activity [as (dFluo./dt)/OD] for cultures in individual wells. OD values were blanked by subtracting the OD readings from wells containing only MOPS medium. Average OD values of more than 10 replicates were used for scattering/inner-filter correction of fluorescence as described above. A control strain without the reporter plasmid was assayed simultaneously to correct for intrinsic background fluorescence of bacterial cells. The average fluorescence of the control samples was subtracted from the reporter profiles at each time point. The resulting fluorescence data were smoothed by weighted averaging of the data from each time point with those from the previous and subsequent time points. For the CFP reporter profiles that display higher noise values, the data were smoothed with a moving average of five time points, including the preceding two time points and the subsequent two time points.

Considering that the data contain unequal time intervals, the first derivative of fluorescence was calculated numerically by differentiating the second-order Lagrange interpolating polynomial using the following equation:

$$\begin{array}{l} f\prime (t_{i})=\frac{t_{i}-t_{i+1}}{\left(t_{i-1}-t_{i}\right)\left(t_{i-1}-t_{i+1}\right)}f\left(t_{i-1}\right)+\frac{2t_{i}-t_{i-1}-t_{i+1}}{\left(t_{i}-t_{i-1}\right)\left(t_{i}-t_{i+1}\right)}f\left(t_{i}\right)+\frac{t_{i}-t_{i-1}}{\left(t_{i+1}-t_{i-1}\right)\left(t_{i+1}-t_{i}\right)}f\left(t_{i+1}\right) \end{array}$$

in which *f* (*t*_*i*_) represents the fluorescence at the *i*^th^ time point. The first derivatives were normalized to the OD for calculation of the promoter activity.

### Measurement of YFP/CFP maturation time.

The maturation time of fluorescence reporter protein was determined by measurement of the fluorescence level present after translation was inhibited by the use of chloramphenicol (Cm) or erythromycin (Ery). YFP expression was induced by phosphate starvation, and 15 μl of chloramphenicol (5.7 mg/ml) was added to 250 μl of bacterial culture 70 min after the induction (final Cm concentration, ~320 μg/ml). Fluorescence was then repeatedly measured every 2 to 2.5 min. After subtraction of background fluorescence from a strain lacking the reporter, the fluorescence was fitted with a first-order kinetic model and the half-life was calculated as the YFP maturation time. The first derivative was derived from the fitted curve. Because the pRG381 CFP reporter plasmid carries a Cm resistance gene, erythromycin (final concentration, 250 μg/ml) was used to inhibit CFP synthesis for maturation time determination.

### Model of PhoB dephosphorylation.

Dephosphorylation dynamics was examined using a simple Michaelis-Menten kinetic model. The binding between PhoB~P and PhoR is described as a fast equilibrium with the constant *K*_*d*_, and the subsequent dephosphorylation is rate-limiting with the rate constant *k*_*p*_. The off-rate constant for the binding is set at 0.5 s^−1^, much larger than *k*_*p*_; thus, in most of the parameter spaces modeled, the Michaelis constant *K*_*m*_ is approximately equal to *K*_*d*_. To model the steady-state PhoB~P level in the absence of a stimulus, all of the PhoR molecules are assumed to function as phosphatase whereas PhoB is phosphorylated by nonspecific sources, such as acetyl phosphate or noncognate HKs, with a first-order rate constant *k*. The rate of PhoB~P change is calculated as follows:

$$\frac{d\left[\text{RR}\;∼\text{P}\right]}{dt}=k*[\text{RR}]-k_{p}*{\left[\text{HK}\right]}_{\text{total}}*\frac{\left[\text{RR}\;∼\text{P}\right]}{K_{m}+\left[\text{RR}\;∼\text{P}\right]}$$

At the steady state, the rate is equal to zero, giving the following equation:

$$\frac{k_{p}}{k}=\frac{{\left[\text{RR}\right]}_{\text{total}}}{{\left[\text{HK}\right]}_{\text{total}}}*\frac{\left(p+K_{m}/{\left[\text{RR}\right]}_{\text{total}}\right)\left(1-p\right)}{p}$$

in which *p* is the PhoB~P fraction and *p* = [RR~P]/[RR]_total_. Steady-state PhoB~P levels in the OFF state were derived from the equation above using indicated parameter values.
