# Supplementary material for: Quantitative Kinetic Analyses of Shutting Off a Two-Component System
Source: mBio. 2017 May 16;8(3):e00412-17. doi: 10.1128/mBio.00412-17 (PMC5433096; doi:10.1128/mBio.00412-17)
Supplement: FIG S1 [file mbo003173306sf1.pdf]

FIGURE S1

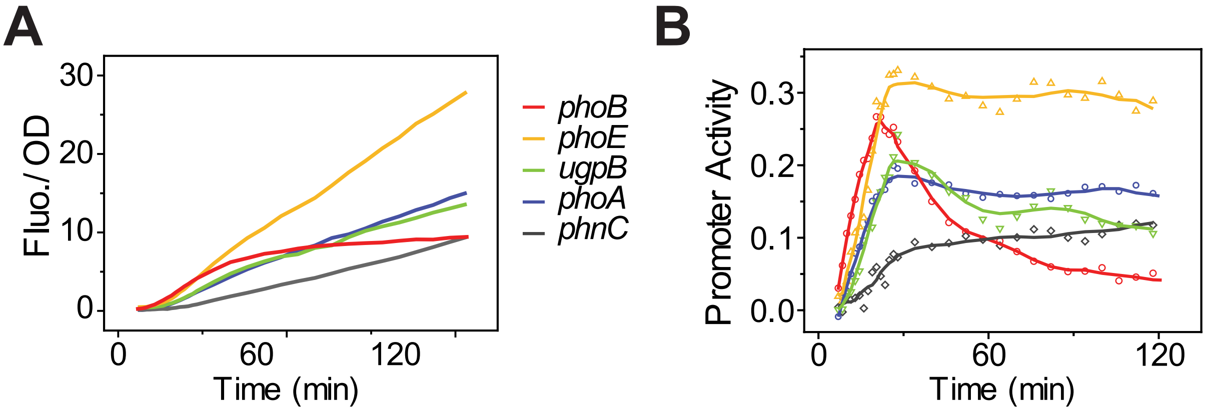

FIG S1 Temporal dynamics of YFP reporter activation illustrated by O.D.-normalized cellular fluorescence (A) and the first derivative of fluorescence (B). WT strains carrying the following reporter plasmids: pJZG202 (*phoB*), pRG347 (*phoE*), pRG346 (*ugpB*), pRG161 (*phoA*) or pRG162 (*phnC*) were assayed for Pi starvation response. Data represent averages of 11 individual wells for each reporter strain. Solid lines represent smoothed data calculated from adjacent averages.
