# Supplementary material for: Quantitative Kinetic Analyses of Shutting Off a Two-Component System
Source: mBio. 2017 May 16;8(3):e00412-17. doi: 10.1128/mBio.00412-17 (PMC5433096; doi:10.1128/mBio.00412-17)
Supplement: FIG S2 [file mbo003173306sf2.pdf]

FIGURE S2

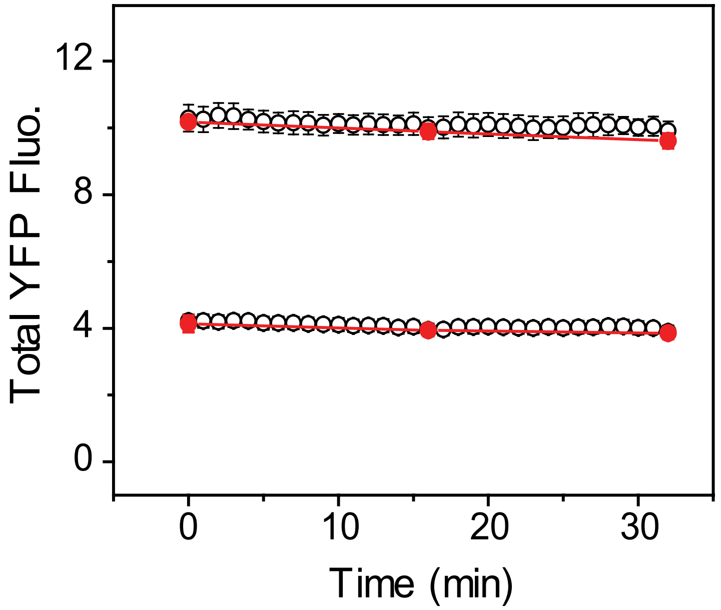

FIG S2 Constant YFP fluorescence after continuous reading. Bacteria with constitutive expression of YFP (BW25141/pRG278) were resuspended in chloramphenicol-containing MOPs media (100 µg/ml) with O.D.s at 0.03 and 0.08 followed by repeated reading once per minute (black circles). Control wells contained identical amount of bacteria but were only read three times at 0, 16 and 32 min (red circles). Frequent reading, or frequent exposures to excitation light, did not significantly alter the total fluorescence of cells, suggesting that there is no significant photobleaching of YFP under experimental conditions.
