# Supplementary material for: Quantitative Kinetic Analyses of Shutting Off a Two-Component System
Source: mBio. 2017 May 16;8(3):e00412-17. doi: 10.1128/mBio.00412-17 (PMC5433096; doi:10.1128/mBio.00412-17)
Supplement: FIG S3 [file mbo003173306sf3.pdf]

FIGURE S3

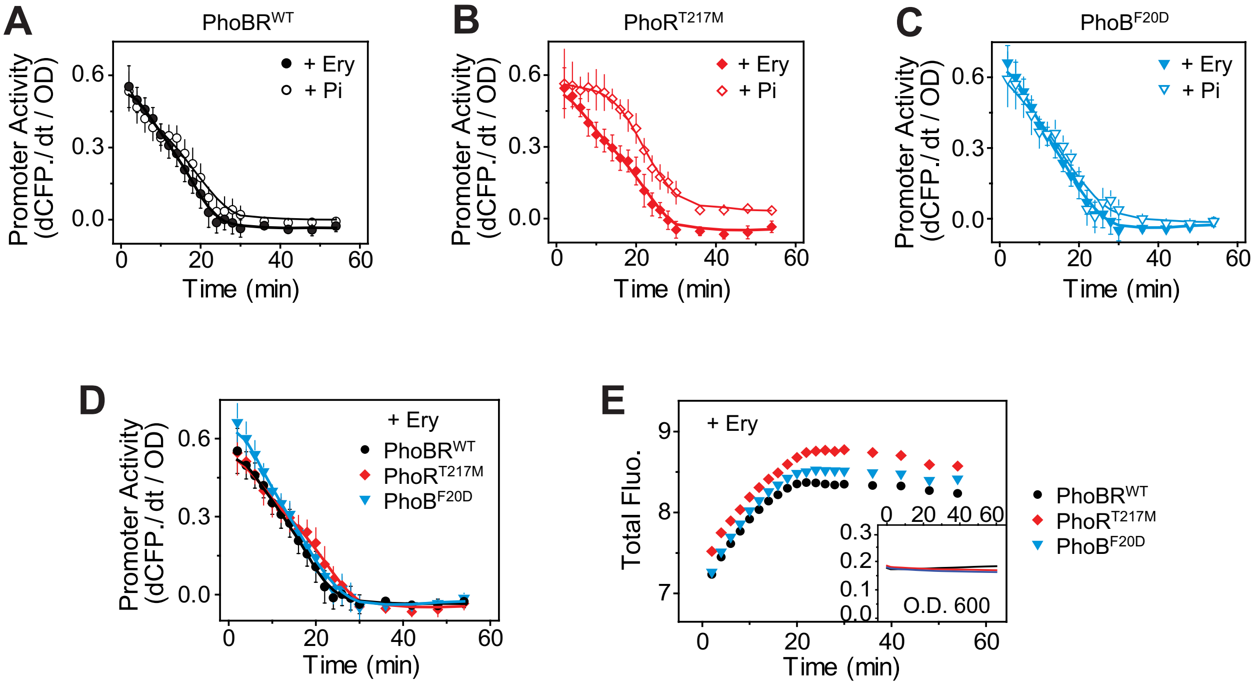

FIG S3 Comparison of shut-off dynamics with CFP maturation kinetics. Decrease of promoter activities due to Pi addition (open symbols) or inhibition of protein synthesis by erythromycin (solid symbols) were compared for RU1823 (A), RU1825 (B) and RU1826 (C). An IPTG concentration of 5 μM was used for steady expression of PhoB. CFP maturation kinetics appeared almost identical for three strains (D and E). Exponential fit of promoter activities in D (Ery treated samples) gives similar maturation half-times as the following: RU1823 (WT), 9.2 min; RU1825 (PhoR^{T217M}), 8.4 min; RU1826 (PhoB^{F20D}), 8.8 min. Average of the three gives the maturation half-time for CFP: 8.8 min. Data are shown as mean ± SD from 11 individual wells.
