# Supplementary material for: Quantitative Kinetic Analyses of Shutting Off a Two-Component System
Source: mBio. 2017 May 16;8(3):e00412-17. doi: 10.1128/mBio.00412-17 (PMC5433096; doi:10.1128/mBio.00412-17)
Supplement: FIG S4 [file mbo003173306sf4.pdf]

FIGURE S4

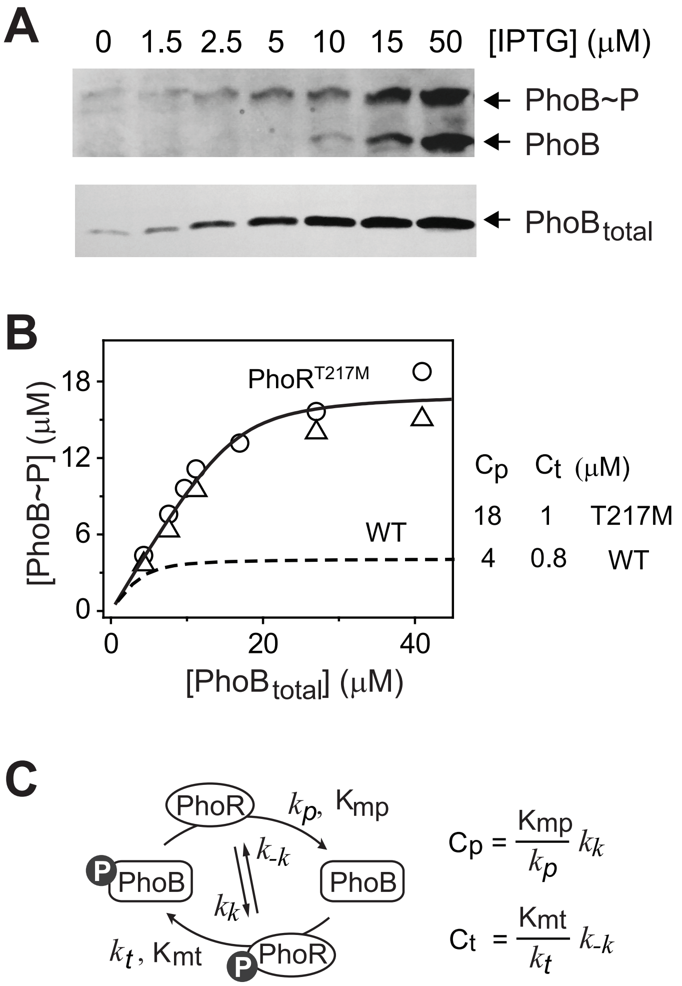

FIG S4 Phosphorylation profiling of RU1825 ($PhoR^{T217M}$). (A) Phosphorylation (upper) and total expression levels (lower) of PhoB were analyzed at different IPTG concentrations. Samples were harvested ~90 min after Pi-depletion and prepared as described before (23). (B) Dependence of PhoB~P levels on PhoB concentrations. Total PhoB concentrations were determined as described previously (10). Phosphorylation levels were calculated as the product of total PhoB concentration and phosphorylation percentages derived from Phos-tag gels. Circles and triangles are data from two independent experiments. The solid line indicates the best fit with the model shown in (C) while the dashed line represents the simulated WT phosphorylation using *Cp* and *Ct* values determined previously (23). Michaelis-Menten kinetic parameters were used to describe the phosphotransfer and dephosphorylation reactions while the steady state levels of PhoB~P are determined by the composite parameters *Cp* and *Ct* (see Text S1). It appears that $PhoR^{T217M}$ has a similar *Ct* value as the WT but a larger *Cp* value, resulting in a higher steady-state phosphorylation level than WT. The high value of *Cp* is unlikely due to an increased K_m because the change of affinity between PhoB and PhoR would simultaneously alter both *Cp* and *Ct.* Given the position of the T217 residue and its conserved role in phosphatase activity in the HisKA family of proteins (5), an ~4.5-fold higher *Cp* for $PhoR^{T217M}$ is most consistent with an ~4.5-fold lower k_p.
