# Supplementary material for: Quantitative Kinetic Analyses of Shutting Off a Two-Component System
Source: mBio. 2017 May 16;8(3):e00412-17. doi: 10.1128/mBio.00412-17 (PMC5433096; doi:10.1128/mBio.00412-17)
Supplement: TEXT S1 [file mbo003173306s1.pdf]

TEXT S1

Model of PhoB phosphorylation and dephosphorylation. To characterize the kinetic parameters of PhoR^{T217M}, the model shown in Fig. S4C was used to fit the phosphorylation profiling data. The model is first described in Batchelor et al (1) and has been used to derive the parameters for the wild type and PhoB^{F20D} (2). Michaelis-Menten kinetic parameters are used to describe the phosphotransfer and dephosphorylation reactions, respectively (Fig. S4C). Steady state RR~P levels at different total concentrations of RR are described by the following equation:

$$[RR \sim P] \approx \frac{1}{2}\left(C_t + C_p + [RR]_{total}\right) - \frac{1}{2}\sqrt{\left(C_t + C_p + [RR]_{total}\right)^2 - 4C_p[RR]_{total}},$$

in which C_p and C_t are composite parameters as shown in Fig. S4C. When $[RR]_{total} >> C_p$, C_t or $[HK]_{total}$, RR~P level is approximately equal to C_p, suggesting a saturation of RR~P level independent of $[RR]_{total}$. Both PhoB^{F20D} and PhoR^{T217M} displayed higher PhoB~P level at high concentrations of total PhoB concentration than that of WT, indicating higher C_p values. Concentration-dependent RR~P levels determined from the phosphorylation profiling data can be fit with the above equation to derive the values of C_p and C_t. As shown in Fig. S4B, PhoR^{T217M} has a similar C_t value but a high C_p value in comparison to the WT. High value of C_p is unlikely due to an increased K_m because a change of affinity between PhoB and PhoR would likely alter both C_p and C_t simultaneously. Decrease of k_p or increase of k_k can both elevate the value of C_p. Because the conserved T or N residue at this position of the HisKA family of HKs has been suggested important for the phosphatase activity (3), a ~4.5 fold increase of C_p value for PhoR^{T217M} was all attributed to a ~4.5 fold decrease of k_p.

As described in Materials and Methods, two reactions including the dephosphorylation by the cognate HK and the non-specific phosphorylation were considered for the modeling of the OFF state. The steady state RR~P fraction can be described by the following equation:

$$\frac{k_p}{k} = \frac{[RR]_{total}}{[HK]_{total}} * \frac{(p + K_m/[RR]_{total})(1-p)}{p}$$

in which p is the PhoB~P fraction, $p = [RR{\sim}P] / [RR]_{total}$. For a certain RR~P fraction p, the ratio of k_p/k is proportional to the ratio of RR to HK. Thus, the higher the ratio of RR to HK is, the stronger the phosphatase, or the higher the ratio of k_p/k need to be to maintain a similar RR~P fraction. Solving the above equation gives:

$$p = \frac{[RR \sim P]}{[RR]_{total}} = \frac{1}{2}\left(\sqrt{(K_m/[RR]_{total} + r - 1)^2 + 4 * K_m/[RR]_{total}} - (K_m/[RR]_{total} + r - 1)\right)$$

in which r = $(k_p / k)*([HK]_{total} / [RR]_{total})$. When $K_m/[RR]_{total} \rightarrow 0$ and r >1, expanding the function gives the first order approximation as the following:

$$\frac{[RR \sim P]}{[RR]_{total}} = \frac{1}{r-1} * \frac{K_m}{[RR]_{total}} + \ldots$$

Therefore, at high concentrations of RR when $[RR]_{total} >> K_m$,

$$[RR \sim P] \approx \frac{K_m}{r-1} = \frac{K_m}{\frac{k_p}{k} * \frac{[HK]_{total}}{[RR]_{total}} - 1}$$

RR~P is a constant dependent on the ratio of RR to HK, the ratio of k_p/k and the K_m of the phosphatase activity. As shown in Fig. 7C, RR~P levels saturate at high concentrations of RR. High ratios of k_p/k or low ratios of RR to HK results in low RR~P levels. Similarly, stronger affinity between HK and RR indicates smaller K_m values, thus lower RR~P levels. When the ratio of k_p/k is close to the ratio of RR to HK, the value of r is close to 1 and the first order approximation is no longer sufficient, but general effects of these parameters on RR~P levels maintain.

1. **Batchelor E., Goulian M.** 2003. Robustness and the cycle of phosphorylation and dephosphorylation in a two-component regulatory system. Proc. Natl. Acad. Sci. USA **100:**691-696.
2. **Gao R., Stock A. M.** 2013. Probing kinase and phosphatase activities of two-component systems *in vivo* with concentration-dependent phosphorylation profiling. Proc. Natl. Acad. Sci. USA **110:**672-677.
3. **Huynh T. N., Stewart V.** 2011. Negative control in two-component signal transduction by transmitter phosphatase activity. Mol. Microbiol. **82:**275-286.
