# Supplementary material for: Quantitative Kinetic Analyses of Shutting Off a Two-Component System
Source: mBio. 2017 May 16;8(3):e00412-17. doi: 10.1128/mBio.00412-17 (PMC5433096; doi:10.1128/mBio.00412-17)
Supplement: TABLE S1 [file mbo003173306st1.pdf]

TABLE S1 Strains and plasmids used in this study.

Strains / plasmids	Relevant characteristics	Reference / source
Strains *E. coli*		
DH5α	General cloning strain	Invitrogen
BL21(DE3)	F^-, *omp*T, *hsd*S_B(r_B-, m_B-), *dcm*, *gal*, λ(DE3)	Novagen
BW25113	Wild-type, *lacI*^{q} *rrnB*$_{T14}$ Δ*lacZ*$_{WJ16}$ *hsdR514* Δ*araBA-D*$_{AH33}$ Δ*rhaBAD*$_{LD78}$	(1)
BW25141	*pir rrnB3* Δ*lacZ4787 hsdR514* DE(*araBAD*)567 DE(*rhaBAD*)568 Δ*phoBR580*	(2)
RU1616	LAC, Φ(Δ*phoBp* P_{lac}-*phoBR*) in BW25113	(3)
RU1621	Δ*phoBR* in BW25113 derivative	(3)
RU1823	P_{trc}-*phoBR, attHK::*pRG351 in RU1621	This study
RU1825	P_{trc}-*phoBR*^{T217M}*, attHK::*pRG352 in RU1621	This study
RU1826	P_{trc}-*phoB*^{F20D}*R, attHK::*pRG330 in RU1621	This study
Plasmids		
pAH144	CRIM plasmid for integration at *attHK* site, Sp^r	(2)
pRG161	*PphoA-mYFP* in pJZG146, Sp^r	(4)
pJZG202	*PphoB-mYFP* in pJZG146, Sp^r	(5)
pRG162	*PphnC-mYFP* in pJZG146, Sp^r	(5)
pRG278	*Ptet-yfp* in pAH63, Km^r	(4)
pRG346	*PugpB-mYFP* in pJZG146, Sp^r	(5)
pRG347	*PphoE-mYFP* in pJZG146, Sp^r	(5)
pRG329	*lacIq & P_{trc}* promoter in pAH144, Sp^r	This study
pRG330	P_{trc}-*phoB(F20D)phoR* in pRG329 for CRIM integration, Sp^r	This study
pRG351	P_{trc}-*phoBR* in pRG330 for CRIM integration, Sp^r	This study
pRG352	P_{trc}-*phoBR*^{T217M} in pRG330 for CRIM integration, Sp^r	This study
pRG381	*PphoA-CFP* in pRG22, Cm^r	(5)

1. **Datsenko K. A., Wanner B. L.** 2000. One-step inactivation of chromosomal genes in *Escherichia coli* K-12 using PCR products. Proc. Natl. Acad. Sci. U S A **97:**6640-6645.
2. **Haldimann A., Wanner B. L.** 2001. Conditional-replication, integration, excision, and retrieval plasmid-host systems for gene structure-function studies of bacteria. J. Bacteriol. **183:**6384-6393.
3. **Gao R., Stock A. M.** 2013. Probing kinase and phosphatase activities of two-component systems *in vivo* with concentration-dependent phosphorylation profiling. Proc. Natl. Acad. Sci. USA **110:**672-677.
4. **Gao R., Stock A. M.** 2013. Evolutionary tuning of protein expression levels of a positively autoregulated two-component system. PLoS Genet. **9:**e1003927.
5. **Gao R., Stock A. M.** 2015. Temporal hierarchy of gene expression mediated by transcription factor binding affinity and activation dynamics. mBio **6:**e00686-00615.
